# Effects of water saving and nitrogen reduction on the yield, quality, water and nitrogen use efficiency of *Isatis indigotica* in Hexi Oasis

**DOI:** 10.1038/s41598-021-04585-x

**Published:** 2022-01-11

**Authors:** Xiucheng He, Huizhen Qiu, Kuizhong Xie, Yucai Wang, Juan Hu, Fuqiang Li, Jing An

**Affiliations:** 1 College of Resources and Environmental Sciences, Gansu Agricultural University, Lanzhou, 730070 China; 2 College of Mechanical and Electrical Engineering, Gansu Agricultural University, Lanzhou, 730070 China; 3 Gansu Provincial Key Laboratory of Aridland Crop Science, Gansu Agricultural University, Lanzhou, 730070 China; 4 Potato Research Institute, Gansu Academy of Agricultural Sciences, Lanzhou, 730070 China; 5 College of Water Resources and Hydropower Engineering, Gansu Agricultural University, Lanzhou, 730070 China; 6 Northeast Institute of Geography and Agroecology, Chinese Academy of Sciences, Changchun, 130102 China

**Keywords:** Ecology, Ecology, Agroecology, Plant sciences, Plant ecology

## Abstract

*Isatis indigotica* planting is the backbone of the medicinal industry in Hexi Oasis, Gansu. In order to solve the problems insufficient water resources and excessive application of nitrogen fertilizer in this area, this paper explored the irrigation and nitrogen levels that can meet the multiple goals of *Isatis indigotica*. The two-factor split-plot field experiment (2018‒2019) was conducted in Minle County, Gansu Province, China, which contains 9 treatments. There were three levels of irrigation water: W1(low), W2(medium), and W3(high). The soil moisture contents were 60–70%, 70–80%, and 80–90% of the field water-holding capacity, respectively. The nitrogen application rate was classified into three levels, N1(low), N2(medium) and N3(high), which were 150, 200 and 250 kg N/ha, respectively. The standard local irrigation water amount and nitrogen application rate corresponded to W3N3. The results showed that the yield of *Isatis indigotica* increased first and then decreased with the increase of irrigation amount and nitrogen application rate, the yield of W2N2 is 12.2–17.1% higher than that of W1N1, the yield of W3N3 was 12.1–17.5% lower than that of W2N2. Saving water and reducing nitrogen can improve the quality of *Isatis indigotica*, compared with W3N3, the indigo, indirubin, (R,S)-epigoitrin and polysaccharides of W2N2 increased by 4.5–5.9%, 2.7–3.1%, 5.2–6.0%, and 1.8–2.1%, respectively. With the increase of nitrogen application rate, the water use efficiency (WUE) first increased and then decreased, as the irrigation volume increases, WUE decreases. Compared with W3N3, the WUE of W2N2 increased by 24.3–27.2%. With the increase of water input, the nitrogen fertilizer use efficiency (NUE) first increased and then decreased, as the nitrogen application rate increases, NUE decreases. Compared with W3N3, the NUE of W2W2 increased by 31.8–34.5%. Therefore, W2N2 can improve quality and increase water and nitrogen utilization efficiency on the basis of ensuring yield.

## Introduction

*Isatis indigotica* is a staple of Chinese medicine. The root of the medicine is Radix isatidis, which has the effects of clearing heat and detoxifying, cooling the blood and relieving sore throat, and has antibacterial and antiviral activities^1^. Minle County in Gansu Province is located at the northern foot of the Qilian Mountains and in the middle of the Hexi Corridor. It is the first county in China to be awarded the title of “Hometown of Radix Isatidis” by the Ministry of agriculture^2^. The root strips of *Isatis indigotica* produced in this area are hypertrophic, whitish, and powdery, with high medicinal value. The *Isatis indigotica* planting area has been larger than 6,667 hm^2^ for many years, with an annual yield of approximately 30,000 tons, it has become an important industry for local farmers to increase income and agricultural efficiency. Irrigation and nitrogen application are key means to regulate crop growth. The scarcity of water resources for irrigation and low fertilizer utilization are common problems in China’s agricultural production, the fertilizer nitrogen use efficiency (NUE) for the main crops in China is only 28–41%, which is far lower than the world average value of 40–60%^3,4^. However, local farmers generally suffer from the problem of excessive nitrogen application. Excessive nitrogen fertilizer input far exceeds crop growth requirements, reducing crop yield and nitrogen fertilizer use efficiency, resulting in waste of nitrogen fertilizer resources and higher production costs. At the same time, the nitrogen that exceeds the absorption range of crops accumulates in In the soil, it is easy to cause adverse effects such as soil compaction and environmental pollution^5^, which threatens the ecological security of Qilian Mountain. The Hexi Oasis is an area with severe water shortages. However, flood irrigation is common among local farmers, which not only reduces crop yields and quality, but also reduces water and nitrogen use efficiency and exacerbates water shortages^6–8^. It is necessary to study the changes in the yield and quality of *Isatis indigotica* and its WUE and NUE under water saving and nitrogen reducing cultivation conditions in Hexi Oasis, this would be beneficial to implementing a water-saving, low-nitrogen, environmentally friendly and efficient way to produce *Isatis indigotica*, this is conducive to the realization of the healthy and sustainable development of the *Isatis indigotica*.

At present, many scholars have conducted research on the effects of water-saving and nitrogen-reducing practices on major food crops^9–11^. The results have shown that compared with traditional irrigation and fertilization methods, water-saving and nitrogen-reducing methods can increase crop yield and effectively improve WUE and NUE. Tavakkoli et al^12^ showed that water saving and nitrogen reduction can improve the yield, WUE and NUE on wheat. Yang et al^13^ showed that both water saving and nitrogen reduction release nitrogen fertilizer management were effective in maintaining rice yield, increasing nitrogen recovery and reducing nitrogen losses from paddy fields, it can provide technical guidance for high-yield and high-efficiency cultivation of rice. Horst et al^14^ showed that when reduce irrigation and reduce nitrogen application, cotton grows vigorously with an optimal plant structure, which significantly promotes the transfer of dry matter to reproductive organs. In addition, the number of effective bolls, the quality of single bolls and the lint content increase; the yield reaches a maximum; and the WUE and NUE improve, respectively. Therefore, reasonable irrigation and nitrogen application are the keys to high crop yield. Optimizing the water and nitrogen management system used in the production of *Isatis indigotica*, reducing water waste and production costs, improving WUE and NUE, and developing water-saving, nitrogen-reducing, high-efficiency and environmentally friendly production models are of vital importance to the sustainable development of agriculture.

During the past, scholars' research on water savings and nitrogen reduction has focused mostly on major food crops such as wheat, corn, and cotton. Research on *Isatis indigotica* has focused mostly on regulated deficit irrigation, fertilizer ratios, and cultivation methods^15–17^. There have been few research reports on the effects of water-saving and nitrogen-reduction practices on the yield and quality of *Isatis indigotica*. Based on *Isatis indigotica* production with high irrigation water amounts and nitrogen application rates by local farmers, this paper studied the effects of different irrigation water amounts and nitrogen application rates on the yield and quality of *Isatis indigotica* and its WUE and NUE. The optimal combination of irrigation water amount and nitrogen application rate to achieve high yield and high-quality *Isatis indigotica* with high WUE and NUE was identified. This study provides a scientific basis for water-saving, low-nitrogen production modes for *Isatis indigotica* in Hexi Oasis.

## Materials and methods

### Experimental site

**Figure 1:**
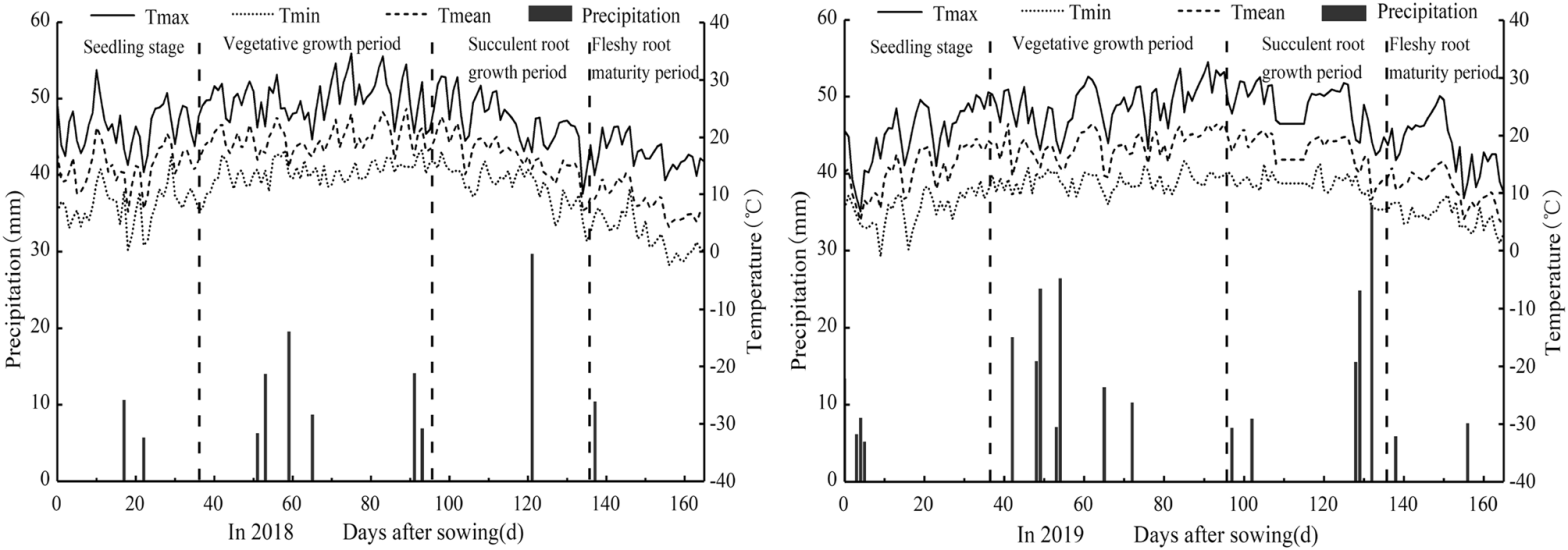
Distribution of monthly rainfall at the experimental station in 2018 and 2019.

A field experiment was conducted at the Yimin Irrigation Experimental Station (East longitude 100°43′, North latitude 38°39′) in Minle County, Gansu Province, in 2018 and 2019. The experimental station has an altitude of 1970 m and a continental desert steppe climate. The annual average temperature is 6 °C, and the annual average precipitation ranges from 183–285 mm, mainly falling from July to September, with an average annual evaporation of 1015 mm^18^. The distribution of monthly rainfall at the experimental station during the test year is shown in Fig. 1.

Use a ring knife to determine the bulk density of the soil, use a pH tester to determine the pH value of the soil, use NaHCO3 extraction-molybdenum antimony colorimetric method to determine available phosphorus, atomic absorption spectrophotometry was used to determine available potassium, Kjeldahl distillation method was used to determine available nitrogen. The basic properties of the experimental field soil are shown in Table 1.

**Table 1:** Physical and chemical properties of the experimental field soil.

Year	Soil layer (cm)	pH	Organic matter (g/kg)	Available nitrogen (mg/kg)	Available phosphorus (mg/kg)	Available potassium (mg/kg)	Bulk density (g/cm^3^)
2018	0–20	7.18	13.86	53.26	8.73	116.16	1.38
2019	0–20	7.21	13.25	55.32	8.68	110.23	1.41

### Experimental materials

In the experiment, large, plump and uniform seeds of *Isatis indigotica* were provided by the local Chinese herbal medicine cooperative. Gansu Provincial Key Laboratory of arid land Crop Science undertook the formal identification of the plant material used in the study. The seed purity was 96.0%, the thousand-grain weight was 9.956 g, the clarity was 88.6%, and the germination rate was 87.6%. The nitrogen fertilizer used in the experiment was urea produced by Gansu Liuhua (Group) Co., Ltd, with a pure nitrogen content of 46%. Superphosphate was used as the phosphate fertilizer produced by Yunnan Gejiu Datong Phosphorus Chemical Industry Co., Ltd, with a P_2_O_5_ content of 16%. Potassium sulfate was used as potash fertilizer produced by Shandong Huali Fertilizer Co., Ltd, with K_2_O ≥ 52%. The distance between the emitters of the drip irrigation belts was 30 cm, the emitter flow was 2.5 L/h, and the normal irrigation pressure was 0.1 MPa. The colour of the agricultural mulch was white, and it had a width of 120 cm and a thickness of 0.08 mm.

### Experimental design

A two-factor, split-plot design for the irrigation water amount (main factor) and nitrogen application rate (sub factor) was adopted. The three levels of irrigation water were set as W1, W2, and W3, and the soil moisture contents were 60%-70%, 70–80% and 80–90% of the field water-holding capacity, respectively. The nitrogen application rates were set as N1, N2 and N3, which were 150, 200 and 250 kg N/ha, respectively. W3 and N3 represent the conventional local irrigation water amount and nitrogen application rate, respectively. Overall, there were 9 treatments with three replicates, constituting a total of 27 plots. Each plot had a length of 8 m, a width of 3.75 m, and an area of 30 m^2^. The effective planting area of the experiment was 810 m^2^. The description of treatments is shown in Table 2.

**Table 2:** Description of treatments.

Irrigation level^a^	Nitrogen level^b^	Treatment abbreviation
High, soil moisture content was 80–90% of the field water-holding capacity (W3)	High, nitrogen application rate: 250 kg/ha (N3)	W3N3(CK)
	Medium, nitrogen application rate: 200 kg/ha (N2)	W3N2
	Low, nitrogen application rate: 150 kg/ha (N1)	W3N1
Medium, soil moisture content was 70–80% of the field water-holding capacity (W2)		W2N3
		W2N2
		W2N1
Low, soil moisture content was 60–70% of the field water-holding capacity (W1)		W1N3
		W1N2
		W1N1

^a^High irrigation level treatment, in which the soil moisture content was 80–90% of the field water-holding capacity. In the medium irrigation level treatment, the soil moisture content was 70–80% of the field water-holding capacity. In the low irrigation level treatment, the soil moisture content was 60–70% of the field water-holding capacity.

^b^High nitrogen level treatment, with a nitrogen application rate of 250 kg/ha. In the medium nitrogen level treatment, the nitrogen application rate was 200 kg/ha. In the low nitrogen level treatment, the nitrogen application rate was 150 kg/ha.

**Figure 2:**
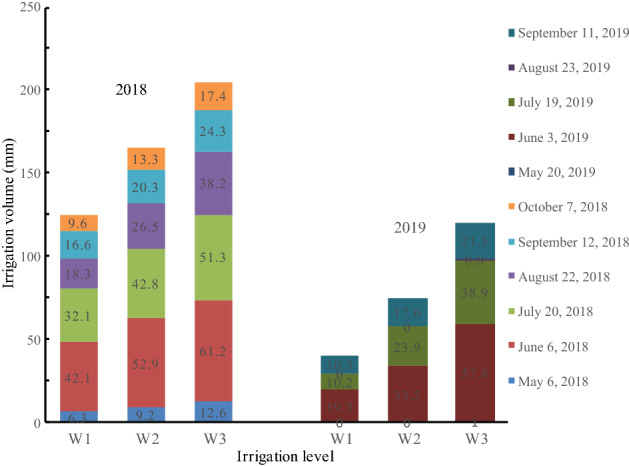
Irrigation schedule in 2018 and 2019.

The experiment uses drip irrigation under the film, and the water meter controls the irrigation volume. The soil moisture control range is 0–60 cm soil layer, and the soil moisture content is measured every 3 days, irrigation was performed when the soil moisture content was lower than the lower limit of the experimental value, and the irrigation amount was determined according to the upper limit of the experimental value. Irrigation time and irrigation amount are shown in Fig. 2.

**Figure 3:**
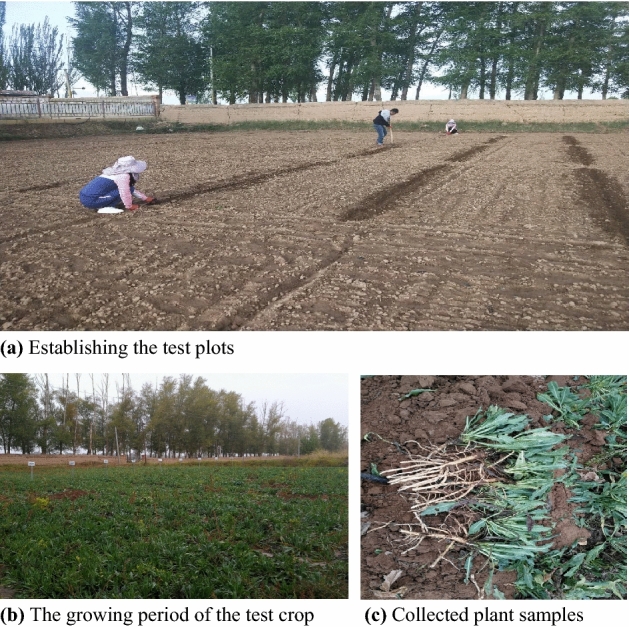
Field experiment.

Tillage was performed in the 0–30 cm soil layer before planting, and weeds were removed manually. In both 2018 and 2019, sowing was performed on May 3rd, and harvesting was performed on October 15th. Flat-land mulched drip irrigation was used. The effective width of the membrane surface was 105 cm, the operational spacing of the membrane surface was 20 cm, the plant spacing was 10 cm, and the average row spacing was 12.5 cm. The sowing rate was 30.0 kg/ha, and the planting density was 800,000 plants/ha. To prevent seepage of water between the treatment plots, the irrigation treatment plots were separated by 60 cm plastic film. In the experimental treatment, nitrogen fertilizer was used as the basal fertilizer according to the nitrogen treatment level, and 350 kg/ha phosphate fertilizer and 200 kg/ha potassium fertilizer were also applied. The field experiment is shown in Fig. 3.

### Measurements and calculations

#### Yield

When the *Isatis indigotica* are harvested, the fresh weight is weighed separately in each plot, and the yield is weighed after natural air-drying.

#### Water use efficiency

The water use efficiency^19^ was calculated as WUE(kg/m^3^) = Y/ET_a_ , where Y is the yield of *Isatis indigotica* (kg/ha) and ET_a_ is the total water consumption during the whole growth period (mm).

ET_a_ = I + P + K + ΔW, where I represents the field irrigation water amount (mm). P is the rainfall during the growth period (mm). K is the amount of groundwater replenishment in the stage (mm), when the groundwater depth is greater than 2.5 m, the K value can be ignored, the groundwater depth in this test is below 20 m, so the groundwater recharge can be regarded as 0. ΔW is the change in soil moisture content before sowing and after harvesting (mm).

ΔW = Soil moisture content before sowing-soil moisture content after harvesting.

#### Nitrogen use efficiency

The nitrogen use efficiency^20^ (NUE, kg/kg) was calculated as the nitrogen accumulation amount/nitrogen fertilizer application rate.

#### The nitrogen content

The nitrogen content of plant samples was determined by the Kjeldahl Method of Nitrogen Determination^21^.

#### Quality

The content of Indigo, Indirubin, and (R,S)-epigoitrin were determined by high performance liquid chromatography^22^. The content of Polysaccharide is determined by phenol-oxidation concentration method^23^.

### Data processing

Statistical analysis software (SPSS 17.0, SPSS Inc., Chicago, IL, USA) was used for the data analysis, the treatment means were compared using Duncan’s multiple-range test at the 0.05 significance level. Origin 2019b (Originlab Corporation, Northampton, USA) was used for the monthly rainfall distribution maps make.

## Results and analysis

### Effects of water and nitrogen treatments on the yield of *Isatis indigotica*

As shown in Table 3, in the two-year experiment, both the water input and the nitrogen application rate had significant effects on the yield of *Isatis indigotica*.

**Table 3:** Variance analysis of traits on the yield of *Isatis indigotica*.

Trait	DF	Variance	SS	MS	F-value	P-value
Yield in 2018	W	2	1,178,560.67	589,280.33	56.13	0.00
	N	2	1,153,888.67	576,944.33	54.96	0.00
	W * N	4	18,925.33	4731.33	0.45	0.47
Yield in 2019	W	2	2,732,828.67	1,366,414.33	95.07	0.00
	N	2	2,255,040.67	1,127,520.33	78.45	0.00
	W * N	4	31,705.33	7926.33	0.55	0.38

**Figure 4:**
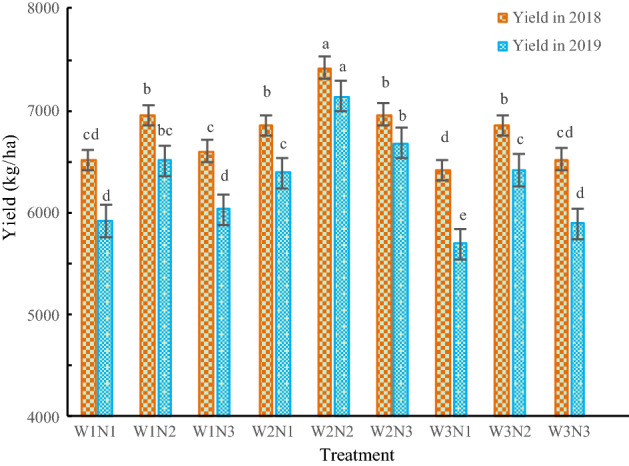
The effects of the different treatments on the yield of *Isatis indigotica*. The values shown are the mean ± SD, n = 3. Asterisks indicate a significant difference at the P ≤ 0.05 level.

As shown in Fig. 4, with increasing water and nitrogen, the yield first increased and then decreased. The interaction between the water input and the nitrogen application rate reached a significant level (P < 0.05). The yield of the W2N2 treatment was the highest, with a value of 7137–7417 kg/ha, followed by that of the W2N3 treatment, with a value of 6679–6962 kg/ha, and the yield of the W3N1 treatment was the lowest, with a value of 5688–6413 kg/ha. The yield of *Isatis indigotica* in the W2N2 treatment increased by 13.7–21.2% compared with that in the W3N3 treatment (P < 0.05).

At the same irrigation level, the yield performance was N2 > N3 > N1. At the levels of W1, W2, and W3, the yield of the N2 treatment increased by 5.3–7.9%, 6.5–6.9%, and 5.0–9.0% compared with those of the N3 treatment, respectively, and the yield of the N3 treatment increased by 1.4–1.9%, 1.5%-4.5%, and 1.7–3.5% compared with those of the N1 treatment, respectively. At the same nitrogen application level, the yield performance was W2 > W1 > W3. At the levels of N1, N2, and N3, the yield of the W2 treatment increased by 6.9–12.4%, 8.3–11.3%, and 6.8–13.5% compared with those of the W3 treatment, respectively, and the yield of the W3 treatment decreased by 1.6–3.9%, 1.5–1.6%, and 1.3–2.4% compared with those of the W1 treatment, respectively.

### Effects of the water and nitrogen treatments on the quality of *Isatis indigotica*

As shown in Table 4, in the two-year experiment, the water input and nitrogen application rate had significant impacts on the contents of indigo, indirubin, (R,S)-epigoitrin and polysaccharide in *Isatis indigotica*.

**Table 4:** Variance analysis of traits the quality of *Isatis indigotica*.

Trait	DF	Variance	SS	MS	F-value	P-value
Indigo in 2018	W	2	0.46	0.23	160.41	0.00
	N	2	0.20	0.10	69.16	0.00
	W * N	4	0.00	0.00	0.38	0.82
Indirubin in 2018	W	2	0.44	0.22	293.07	0.00
	N	2	0.19	0.09	122.60	0.00
	W * N	4	0.02	0.01	7.46	0.00
(R,S)-epigoitrin in 2018	W	2	0.00	0.00	3290.53	0.00
	N	2	0.00	0.00	1378.76	0.00
	W * N	4	0.00	0.00	8.88	0.00
	W	2	22.95	11.47	3374.77	0.00
Polysaccharide in 2018	N	2	10.96	5.48	1611.95	0.00
	W * N	4	0.54	0.13	39.51	0.00
Indigo in 2019	W	2	0.22	0.11	140.19	0.00
	N	2	0.12	0.06	76.80	0.00
	W * N	4	0.00	0.00	1.33	0.30
Indirubin in 2019	W	2	0.37	0.19	241.87	0.00
	N	2	0.16	0.08	105.04	0.00
	W * N	4	0.01	0.00	1.80	0.17
(R,S)-epigoitrin in 2019	W	2	0.00	0.00	18,507.87	0.00
	N	2	0.00	0.00	5944.38	0.00
	W * N	4	0.00	0.00	93.35	0.00
Polysaccharide in 2019	W	2	29.96	14.98	14,191.20	0.00
	N	2	11.94	5.97	5654.75	0.00
	W * N	4	0.34	0.09	80.67	0.00

**Figure 5:**
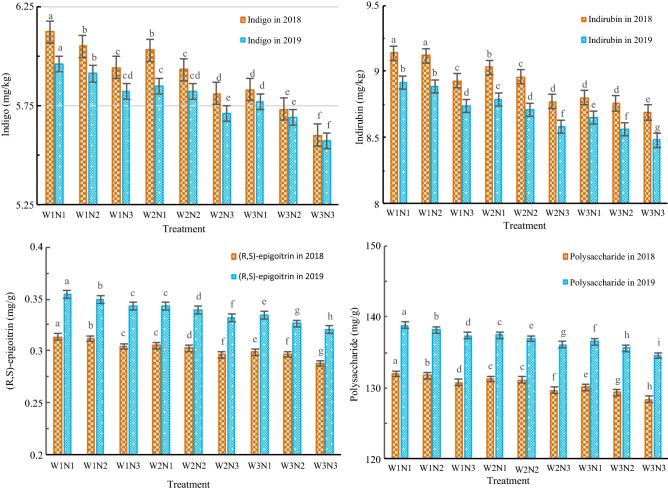
The effects of the different treatments on the quality index of *Isatis indigotica*. The values shown are the mean ± SD, n = 3. Asterisks indicate a significant difference at the P ≤ 0.05 level.

As shown in Fig. 5, The impacts decreased with increasing irrigation amount and nitrogen application rate. Compared with those in the W3N3 treatment, the contents of indigo, indirubin, (R,S)-epigoitrin and polysaccharide in the W2N2 treatment increased by 4.5–5.9%, 2.7–3.1%, 5.2–6.0% and 1.8–2.1%, respectively. At the same irrigation level, the contents of indigo, indirubin, (R,S)-epigoitrin and polysaccharides all decreased in the order N1 > N2 > N3. At the W2 level, the contents of indigo, indirubin, (R,S)-epigoitrin and polysaccharide in the N1 treatment increased by 0.5–1.7%, 0.8–0.9%, 0.8–1.1% and 0.1–0.4%, respectively, compared with those in the N2 treatment. Compared with those in the N3 treatment, the contents of indigo, indirubin, (R,S)-epigoitrin and polysaccharide in the N2 treatment increased by 1.9–2.1%, 1.5–2.2%, 2.1–2.2% and 0.6–1.1%, respectively.

At the same nitrogen level, the contents of indigo, indirubin, (R,S)-epigoitrin and polysaccharides all decreased in the order W1 > W2 > W3. At the N2 level, the contents of indigo, indirubin, (R,S)-epigoitrin and polysaccharides in the W1 treatment increased by 1.5–2.0%, 1.8–2.1%, 3.0–3.1% and 0.4–0.9% compared with those in the W2 treatment, respectively. Compared with those in the W3 treatment, the contents of indigo, indirubin, (R,S)-epigoitrin and polysaccharides of the W2 treatment increased by 2.3–3.5%, 1.8–2.3%, 2.0–4.0% and 1.0–1.4%, respectively.

### Effects of the water and nitrogen treatments on the WUE of *Isatis indigotica*

As shown in Table 5, in the two-year experiment, the water input and nitrogen application rate had significant impacts on the WUE of *Isatis indigotica* (P < 0.05).

**Table 5:** Variance analysis of traits on the WUE of *Isatis indigotica*.

Trait	DF	Variance	SS	MS	F-value	P-value
WUE in 2018	W	2	1.50	0.75	906.59	0.00
	N	2	0.18	0.09	111.17	0.00
	W * N	4	0.00	0.00	0.54	0.71
WUE in 2019	W	2	1.12	0.56	542.70	0.00
	N	2	0.23	0.12	113.03	0.00
	W * N	4	0.00	0.00	0.42	0.79

**Figure 6:**
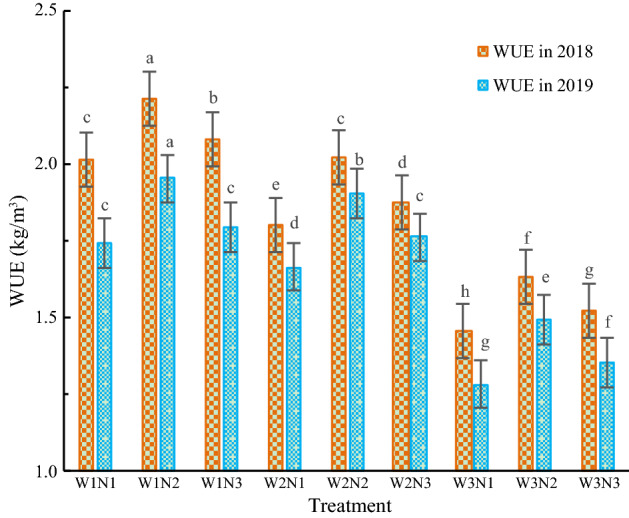
The effects of the different treatments on the WUE of *Isatis indigotica*. The values shown are the mean ± SD, n = 3. Asterisks indicate a significant difference at the P ≤ 0.05 level.

As shown in Fig. 6, WUE decreased with increasing irrigation amount. The WUE first increased and then decreased with increasing nitrogen application rate. The WUE of the W1N2 treatment was the highest, with a value of 1.95–2.21 kg/m^3^, followed by the WUE of the W2N2 treatment, with a value of 1.90–2.02 kg/m^3^. The WUE of the W3N1 treatment was the lowest, with a value of 1.28–1.46 kg/ m^3^. The WUE of the W1N2 treatment increased by 7.8–8.1% compared with that of the W2N2 treatment, and the WUE of the W2N2 treatment increased by 24.3–27.2% compared with that of the W3N3 treatment.

At the same irrigation level, the WUE performance was N2 > N3 > N1. At the W1, W2, and W3 levels, the WUE of the N2 treatment increased by 6.5–8.6%, 7.8–8.1%, and 7.4–10.4% compared with that of the N3 treatment, respectively, and the WUE of the N3 treatment increased by 2.9–3.1%, 3.9–6.0%, and 4.5–5.3% compared with that of the N1 treatment, respectively. Under the same nitrogen application rate level, the WUE performance was W1 > W2 > W3. At the N1, N2, and N3 levels, the WUE of the W1 treatment increased by 5.0–11.7%, 2.8–9.2%, and 2.0–10.9% compared with that of the W2 treatment, respectively, and the WUE of the W2 treatment increased by 24.2–29.5%, 24.3 -27.2%, and 23.5–30.3% compared with that of the W3 treatment, respectively.

### Effects of water and nitrogen treatments on NUE of *Isatis indigotica*

As shown in Table 6, in the two-year experiment, the water input and nitrogen application rate had significant impacts on the nitrogen fertilizer use efficiency (NUE) of *Isatis indigotica* (P < 0.05).

**Table 6:** Variance analysis of traits on the NUE of *Isatis indigotica*.

Trait	DF	Variance	SS	MS	F-value	P-value
NUE in 2018	W	2	0.01	0.00	149.57	0.00
	N	2	0.06	0.03	1103.57	0.00
	W * N	4	0.00	0.00	2.14	0.12
NUE in 2019	W	2	0.00	0.00	51.55	0.00
	N	2	0.04	0.02	521.18	0.00
	W * N	4	0.00	0.00	2.86	0.05

**Figure 7:**
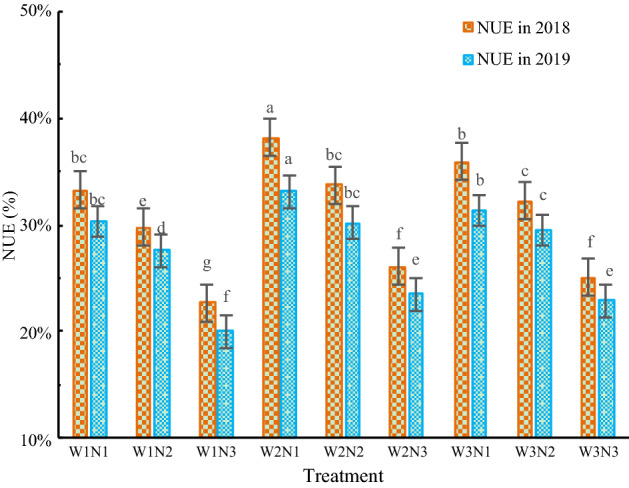
The effects of the different treatments on the NUE of *Isatis indigotica*. The values shown are the mean ± SD, n = 3. Asterisks indicate a significant difference at the P ≤ 0.05 level.

As shown in Fig. 7, With the increase in irrigation amount, NUE increased first and then decreased. With the increase in the nitrogen application rate, NUE decreased gradually. The NUE of the W2N1 treatment was the highest, reaching 33.1–38.1%, followed by those of the W3N1, W1N2, W1N1 and W2N2 treatments, which were in the range of 20.0–35.9%. The differences in NUE among these treatments were not significant. The NUE of the W3N1 treatment was the lowest, at 20.1–22.7%. Compared with that of the W2N2 treatment, the NUE of the W2N1 treatment increased by 9.6–13.0%, and the NUE of the W2N2 treatment increased by 31.8–34.5% compared with that of the W3N3 treatment.

At the same irrigation level, the NUE performance was N1 > N2 > N3. At the levels of W1, W2, and W3, the NUE of the N1 treatment increased by 9.9–11.8%, 9.6–13.0%, and 6.3–11.6% compared with that of the N2 treatment, respectively, and the NUE of the N2 treatment increased by 31.0–37.6%, 28.8–29.2%, and 28.3–28.6% compared with that of the N3 treatment, respectively. At the same nitrogen application level, the NUE performance was W2 > W3 > W1. At the N1, N2, and N3 levels, the NUE of the W2 treatment increased by 5.7–6.1%, 2.5–4.8%, and 2.3–4.1% compared with that of the W3 treatment, respectively, and the NUE of the W3 treatment decreased by 3.4–8.0%, 6.9–8.2%, and 10.5–14.5% compared with that of the W1 treatment, respectively.

### Ethical guideline

The authors confirm that relevant ethical guidelines were followed for plant usage.

### Land permit statement

The experimental land belongs to the Yimin Irrigation Experimental Station, in Minle County, Gansu Province, China.

## Discussion

### Effects of water and nitrogen treatments on the yield of *Isatis indigotica*

Water and nitrogen are the key factors regulating crop production. An appropriate amount of irrigation water and nitrogen guarantees high crop yield, resource conservation and environmentally friendly agricultural development^24–26^. In agricultural production, irrigation and nitrogen application rates are increased in order to increase yield. However, in actual production, crop yield does not increase proportionally with increases in irrigation and nitrogen application, rather, they reflect the law of diminishing returns. Girón et al^27^ reported that continuous mild water-deficit regulation could achieve water-saving and efficient production of crop. Irrigation and nitrogen application have been shown to have a significant interaction effect on yield^28,29^, which is consistent with our results. The yield of *Isatis indigotica* increased first and then decreased with increasing irrigation water amount and nitrogen application rate in this study. When the soil moisture content was reduced to 70–80% of the field water-holding capacity and the nitrogen application rate was reduced to 200 kg/ha, the yield of *Isatis indigotica* was the highest; the yield increased by 13.7–21.2% compared with that of the standard treatment. This shows that appropriate water savings and nitrogen reduction can increase the yield of *Isatis indigotica* and that flood irrigation and excessive nitrogen application hinder high yield of *Isatis indigotica*. Salahi et al^30^ obtained similar results; that is, under proper irrigation and fertilization conditions, high crop yields can be obtained. Eneji et al^31^ showed that moderate irrigation is more conducive to increasing maize yield than excessive irrigation. Buresh et al^32^ showed that moderate nitrogen fertilization is more conducive to increasing crop yield than excessive nitrogen fertilization. Therefore, compared with local farmers' excessive water and nitrogen application (W3N3), water saving practices and nitrogen reduction can increase production.

### Effects of water and nitrogen treatments on the quality of *Isatis indigotica*

Indigo, indirubin and (R,S)-epigoitrin are the main active ingredients in *Isatis indigotica*. Indigo and indirubin are indole compounds, and (R,S)-epigoitrin is a sulfur-containing compound^33^; they are secondary metabolites produced by *Isatis indigotica*^34^. The synthesis and accumulation of secondary metabolites are closely related to the growth environment in which a plant is located, and they tend to be produced in higher quantities when plants are in stressful environments, such as those experiencing drought, barrenness, low temperatures, high salinity, or water logging^35^. Ko et al^36^ reported that the continuous mild or moderate regulation of water deficit during the vegetative growth period and storage root growth period could significantly increase the contents of indigo, indirubin and (R,S)-epigoitrin. The results of this study also showed that at the same nitrogen level, the contents of indigo, indirubin and (R,S)-epigoitrin all decreased with increasing irrigation amount. These results indicate that a low irrigation level is beneficial for improving the quality of *Isatis indigotica* because the stressful environment created by the low irrigation level is helps to increase the contents of secondary metabolites, such as indigo, indirubin and (R,S)-epigoitrin. Lu et al^37^ reported that appropriately reducing the nitrogen application rate resulted in *Isatis indigotica* with higher active ingredient contents. This study showed that at the same irrigation level, indigo, indirubin, (R,S)-epigoitrin and polysaccharides all decreased with increasing nitrogen application rate, indicating that a low nitrogen application rate is beneficial to improve the quality of *Isatis indigotica*. This occurred because the stressful environment created by the low level of nitrogen application helped to increase the contents of secondary metabolites, such as indigo, indirubin and (R,S)-epigoitrin. Polysaccharides are also active components of *Isatis indigotica* and are primary metabolites. When the environment is suitable, plants synthesize proteins, carbohydrates, nucleic acids and other substances needed for their growth through primary metabolism. Ho et al^38^ showed that the polysaccharide content of *Isatis indigotica* was the highest under slight flooding irrigation and moderate nitrogen treatments. This study also showed that compared with the W3 level, the polysaccharide content of the W2 level increased by 0.7–1.4%, and the polysaccharide content of the W1 level increased by 1.5–2.1%, water-saving treatment can increase the polysaccharide content. Compared with the N3 level, the polysaccharide content of the N2 level increased by 0.6–1.1%, and the polysaccharide content of the N1 level increased by 0.9–1.5%, nitrogen reduction treatment can increase the polysaccharide content. The reason may be that the adversity environment of low water and low nitrogen stimulated the accumulation of polysaccharide in *Isatis indigotica*.

In addition, this study found that low irrigation and low nitrogen levels helped to increase the polysaccharide content. This may have occurred because the adverse environment created by the low irrigation and low nitrogen levels stimulated the formation of polysaccharides in *Isatis indigotica*. Therefore, appropriate water and nitrogen reductions can improve the quality of *Isatis indigotica*. Compared with those in the W3N3 treatment, the contents of indigo, indirubin, (R,S)-epigoitrin and polysaccharide in W2N2 increased by 4.5–5.9%, 2.7–3.1%, 5.2–6.0% and 1.8–2.1%, respectively.

### Effects of water and nitrogen treatments on the WUE of *Isatis indigotica*

WUE is an important indicator of water use in crop production. To reduce water resource waste in agricultural production, research on how to achieve high WUE in crops is necessary. Nagaz et al^39^ showed that WUE decreased with increasing irrigation amount and increased with increasing nitrogen application rate. Our study showed that water use efficiency (WUE) first increased and then decreased with the increase of nitrogen application rate, as the irrigation volume increases, water use efficiency (WUE) decreases. When the soil moisture content was 60–70% of the field water-holding capacity, and the nitrogen application rate was 200 kg N/ha, the WUE was the highest. The reason for this discrepancy is that the three nitrogen application rates in our experiment were 150, 200 and 250 kg N/ha, which are much higher than those established by Feng Fuxue et al. When excessive nitrogen is applied, crop growth is hindered, thereby reducing crop water use efficiency. Therefore, reducing water and nitrogen use is beneficial to improving the WUE of *Isatis indigotica*. Compared with W3N3, the water use efficiency of W2N2 is increased by 24.3% to 27.2%. Halford. et al^40^ showed that the peak value for crop yield was not completely consistent with the WUE. Our study showed that the W2N2 treatment corresponded to the maximum yield of *Isatis indigotica* but did not have the highest WUE. Compared with the WUE of the W1N1 treatment, which was the highest, the WUE of the W2N2 treatment was reduced by 4.3–7.7%. Thus, high yield is not consistent with high WUE in the production of *Isatis indigotica*. In general, drip irrigation under mulch greatly reduces the total amount of irrigation water used during the growth period of *Isatis indigotica* and greatly improves the water use efficiency of production. However, due to the limitations of the test conditions, such as the one-time application of granular urea, the NUE of production in this study was relatively low. Therefore, in the later stages, water-soluble nitrogen fertilizer could be applied with a dropper, and the concentration of nitrogen fertilizer could be adjusted according to the growth period of *Isatis indigotica*. It is necessary to further explore the practical significance of and provide operational guidance related to the temporal and spatial effects of water and nitrogen on water saving and nitrogen reduction practices.

### Effects of water and nitrogen treatments on the NUE of *Isatis indigotica*

NUE is another important indicator for crop production. In agricultural production, research on how to achieve high NUE in crops is necessary to reduce production costs and environmental pollution risks. Hatfield et al^41^ showed that the NUE decreased with increasing nitrogen application rate. Zhang et al^42,43^ reported that the NUE first increased and then decreased with increasing irrigation water amount and that excessive irrigation reduced the NUE. Our study showed that the NUE first increased and then decreased with the increase of irrigation amount, with the increase of nitrogen application rate, the NUE decreases. When the soil moisture content was 70–80% of the field water-holding capacity and the nitrogen application rate was 150 kg N/ha, the NUE was the highest. The reason for this discrepancy is that within a certain irrigation range, the NUE increases with the increase of the irrigation amount. When the irrigation is excessive, the growth of the crop is hindered, which is not conducive to the absorption and utilization of nitrogen by the crop. Therefore, using less water and nitrogen is conducive to improving the NUE of *Isatis indigotica*. The NUE of the W2N2 treatment increased by 31.8–34.5% compared with that of the W3N3 treatment. In agricultural production, the goals of achieving high yield, high Water and Nitrogen Use Efficiency, resource and environmental security are contradictory. Further research on the coupling effect and mechanisms of the water-nitrogen interaction is needed.

In summary, appropriate water and nitrogen use reductions can increase yield, improve grain quality, and improve the efficiency of water and nitrogen use in *Isatis indigotica*. excessive irrigation and fertilization will not only reduce crop yield and quality, but also reduce the water and nitrogen use efficiency of crops and cause environmental pollution.

## Conclusions

Water input and nitrogen application have significant effects on the yield, quality, water and nitrogen use efficiency of *Isatis indigotica*. With the increase of water input and nitrogen application rate, the yield increased first and then decreased. Saving water and reducing nitrogen can improve the quality of *Isatis indigotica*. With the increase of nitrogen application rate, WUE first increased and then decreased, as the water input increases, WUE decreases. With the increase of water input, NUE first increased and then decreased. With the increase of nitrogen application rate, NUE decreases. Therefore, W2N2 can improve quality and increase water and nitrogen utilization efficiency on the basis of ensuring yield.
